# Supplementary material for: Nanosecond-resolution photothermal dynamic imaging via MHZ digitization and match filtering
Source: Nat Commun. 2021 Dec 7;12:7097. doi: 10.1038/s41467-021-27362-w (PMC8651735; doi:10.1038/s41467-021-27362-w)
Supplement: Supplementary file 1 — Supplementary Information [file 41467_2021_27362_MOESM1_ESM.pdf]

Supplementary Information

Nanosecond-Resolution Photothermal Dynamic Imaging via MHz Digitization and Match Filtering

Jiaze Yin et al.

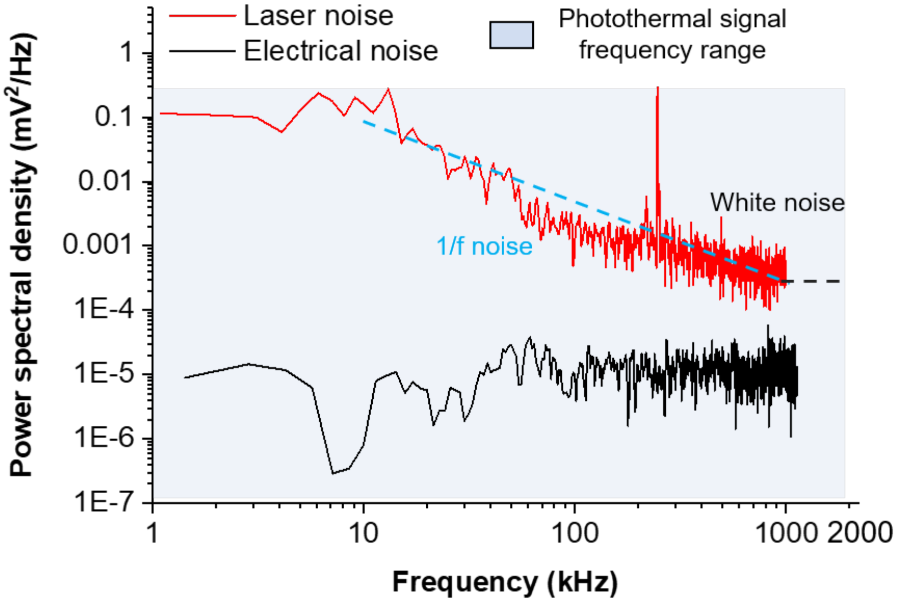

Supplementary Figure 1. System noise spectrum. Laser noise was measured by a broadband photodiode (DET10A, Thorlabs) using the lock-in sweeper function when the IR laser was off and visible laser irradiance 1mW on the detector. The electrical noise is measured at amplifier output with 40dB gain and connected photodiode but the visible laser is off.

Supplementary Note 1 | Improvement of PDI detection sensitivity over the lock-in detection method

We define the SNR of photothermal modulation under pulse excitation as the signal's peak amplitude divided by noise amplitude. With impulse excitation assumed, the signal amplitude S_n at nth harmonics at frequency f_n is proportional to the magnitude of absorber's heat transfer function $H(f_n)$. Taking the analogy to a first-order resistor-capacitor circuit, the $H(f_n)$ is given by:

$$H(f_n) = \frac{1}{1 + j2\pi\tau f_n} \tag{1}$$

where τ is the thermal decay constant. For a given absorber with defined decay constant, its peak amplitude S under impulse excitation with nth correlated harmonics captured is given by the following relation, where A is a scaling factor:

$$S = S_1 + S_2 + \cdots + S_n = A(|H(f_1)| + |H(f_2)| + \cdots + |H(f_3)|) \tag{2}$$

The uncorrelated noise amplitude captured in the same nth harmonics is calculated by $\sqrt{|N_1|^2 + |N_2|^2 + \cdots + |N_n|^2}$, where N_n is the noise amplitude at nth harmonics frequency.

As a result, the SNR is written as:

$$\mathrm{SNR} = \frac{S_1 + S_2 + \cdots + S_n}{\sqrt{|N_1|^2 + |N_2|^2 + \cdots + |N_n|^2}} \tag{3}$$

For lock-in based PHI detection of the first order harmonic, SNR_{LIA} is represented as:

$$\mathrm{SNR_{LIA}} = \frac{S_1}{N_1} \tag{4}$$

We define the signal amplitude's ratio between ith harmonic and first harmonic as $a_i = S_i/S_1$, and noise amplitude's ratio as $b_i = N_i/N_1$ correspondingly. In addition, the amplitude of noise has the relationship of $N_i^2/N_1^2 = f_1/f_i$ for 1/f noise dominant region. The SNR of PDI with nth concurrently detected harmonics is then written as:

$$\mathrm{SNR_{PDI}}(n) = \frac{(1 + a_2 + + \cdots + a_n)}{\sqrt{1 + b_2^2 + \cdots + b_n^2}} \mathrm{SNR_{LIA}} \tag{5}$$

The SNR improvement is subject to the term $(1 + a_2 + + \cdots + a_n)/\sqrt{1 + b_2^2 + \cdots + b_n^2}$. This term depends on the IR modulation frequency and absorber's decay constant collectively.

Based on the above equation, we estimate the SNR improvement factor for photothermal imaging of D=300 nm PMMA particle with a decay constant of 280 ns. With an IR repetition rate at 100 kHz, PDI SNR improvement versus the order of captured harmonics is shown in Fig.S2a. By capturing up to 16 orders of harmonics (1.6 MHz), 5.4 times SNR improvement will be expected. Experimentally, our result shows a 4.3 times improvement, which is close. The discrepancy might be attributed to the non-ideal pulse shape of the actual IR excitation pulse while an ideal IR impulse excitation and reduced 1/f noise at megahertz frequency are assumed.

The SNR improvement factor becomes larger as the signal is modulated at a lower frequency and the duty cycle is small. In such a case, the energy of modulated signal extends to a relatively broadband in the frequency domain, and the fundamental harmonic at low frequency is dominant by strong laser fluctuation noise. For example, under the nanosecond IR excitation with a low repetition rate of 20 kHz, the PDI signal of D=300 nm PMMA particle gains 22 times for expected SNR improvement by capturing 80th harmonics (1.6 MHz) over the single frequency demodulation at 20kHz via lock-in amplifier as shown in Fig. S2b.

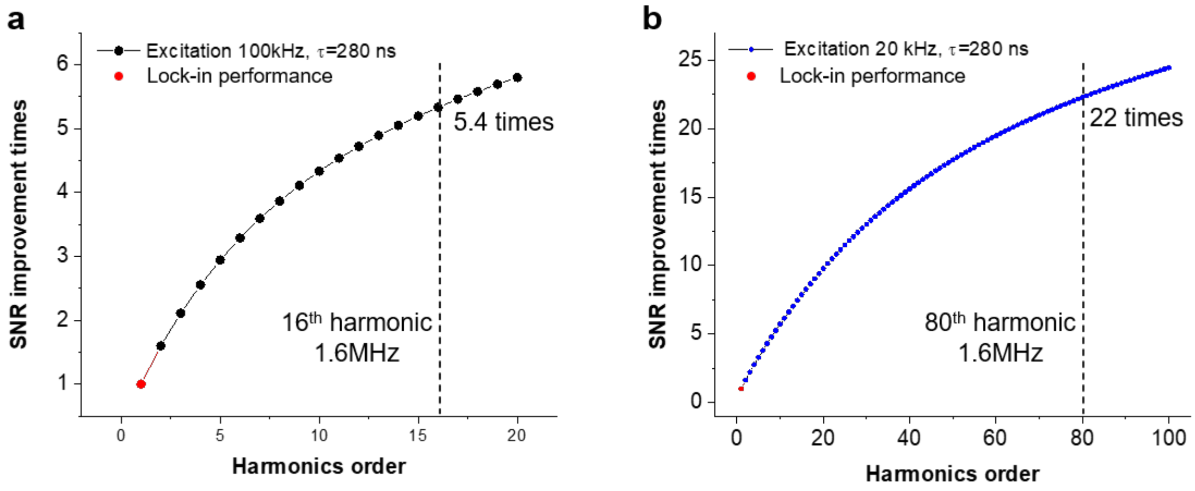

Supplementary Figure 2. Estimated SNR improvement times versus captured harmonics order. (a) SNR improvement factor of photothermal detection D=300 nm PMMA particle with a decay constant of 280 ns under IR repetition rate of 100 kHz. (b) SNR improvement factor of detecting the same sample of (a) under an IR repetition rate of 20 kHz. For the above estimation impulse excitation and 1/f noise model are assumed.

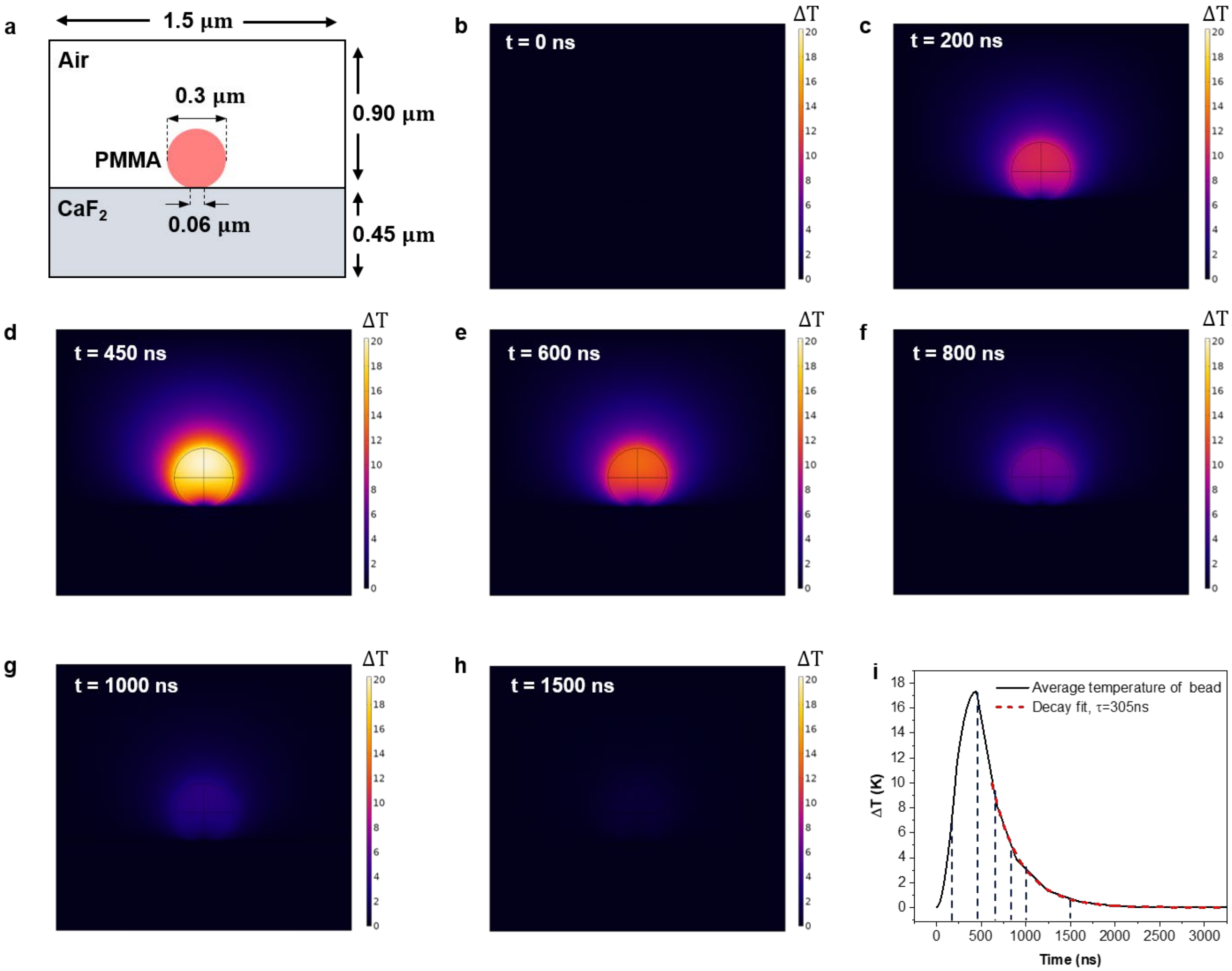

Supplementary Figure 3. Simulation of 300-nm diameter PMMA particle temperature evolvement under pulsed IR heating. (a) Geometric configuration used in the simulation by COMSOL Multiphysics (version 5.5, COMSOL AB, Sweden). Mid-IR heating pulse is set as a symmetric triangle wave with duration of 600 ns and peak intensity of 5.3E-8W/m^2. (b-h) The temperature distribution at 0 ns, 200 ns, 450 ns, 600 ns, 800 ns, 1000 ns, 1500 ns, respectively. (i) The time dependent temperature of PMMA particle and corresponding decay constant τ.

Supplementary Note 2 | PDI of nanoparticles with different size

From the photothermal dynamic model we build, both the temperature rise and decay are strongly related to the time constant mC_s/hS. For spherical particles embedding in a uniform medium, hS can be approximated by $2\pi kD$[1], where k is the medium heat conductivity and D is the particle diameter. As a result, the decay constant is proportional to $r^2\rho C_s/k$. For the particle with the same material and uniform microenvironment, the time constant has an r^2dependency. To validate this relationship, we performed PDI of PMMA particles of different sizes, as shown in Fig. S4a. Besides the photothermal intensity difference, we observed a significant difference in their thermal dynamics. The thermal dynamics and heat flux function of indicated particles in Fig. S3a are shown in Fig. S4b and Fig. S4c. The retrieved decay constant for the 300 nm and 500 nm particles are 290 ns and 540 ns, respectively. For statistical analysis, we fit the decay signal at each pixel and generate a decay constant map, indicating the thermal lifetime, as shown in Fig. S4d. The histogram of the selected area in decay constant map is shown in Fig. S4e, where we observe two peaks representing 300 nm and 500 nm with center value of 280 ns and 495 ns, respectively. From this result, the decay constant scaled for 1.8 times between 300 nm and 500 nm particles, smaller than 2.8 times that is estimated with r^2 dependency. This difference is majorly caused by the influence of variation of substrate-contact area of different particles. CaF_2 substrate has a much larger heat conductivity (9.71 W/(mK)) than air (0.026 W/(mK)).

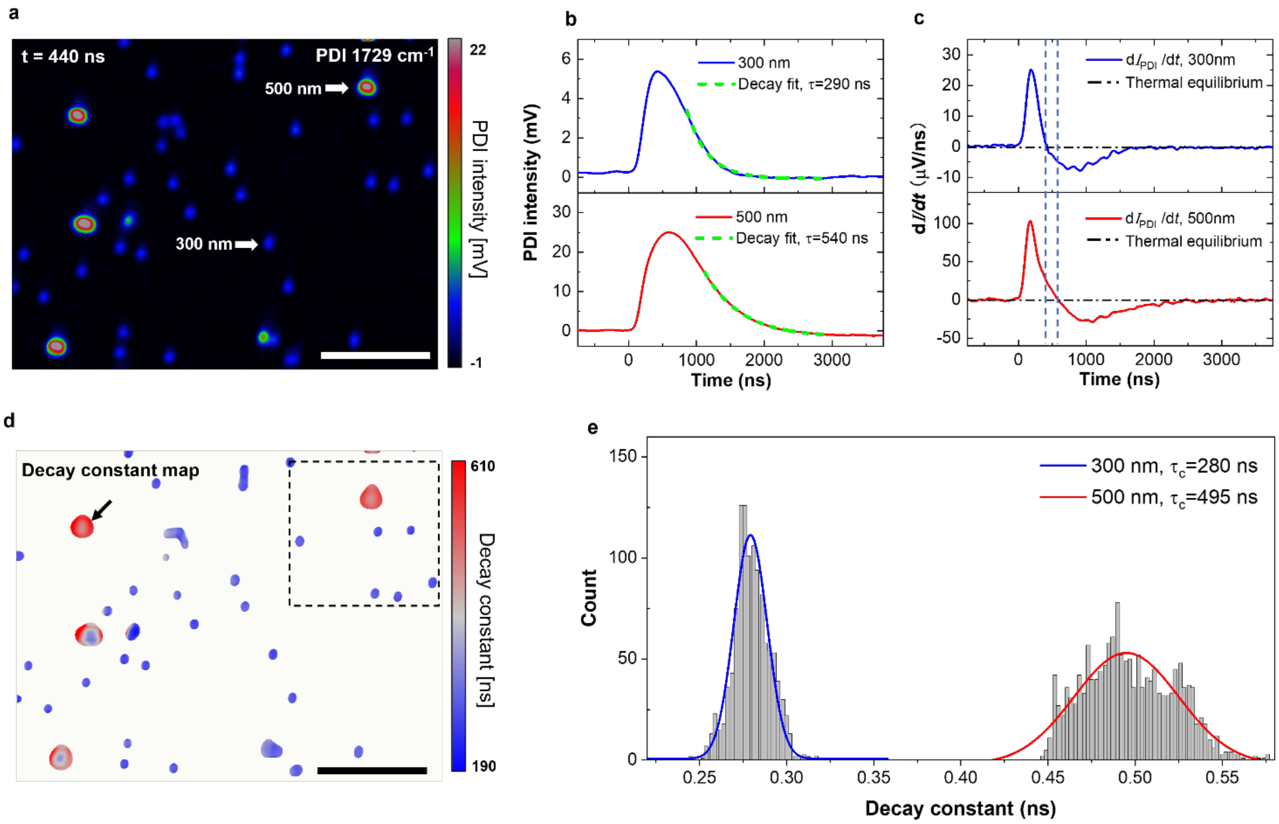

Supplementary Figure 4. PDI of nanoparticles with different size and decay constant map. (a) PDI acquired photothermal intensity image of 300-nm and 500-nm diameter PMMA particles mixture at absorption peak 1729 cm^{-1}. (b) Photothermal dynamics of 300-nm and 500-nm diameter particles. The dash line is the exponential decay fitting with corresponding time constant τ. (c) Time-resolved energy flux function acquired by derivative (b) over time. (d) Decay constant map. Acquired by fitting the temporal signal at every pixel with SNR larger than 20. (e) Histogram of decay constant map of selecting area in (d). The decay constant of 300-nm and 500-nm diameter particles shows two peaks with center at 280 ns and 495 ns, respectively. Pixel dwell time: 200 μs; Probe power on sample: 20 mW; pump power on sample: 4 mW at 1729 cm^{-1}; Scale bars: 5 μm.

Supplementary Note 3 | Transient temperature detection via PDI

With the nanosecond temporal resolution for measuring the complete thermodynamics, PDI enables transient temperature detection. Here, we retrieved the temperature rise for D=500 nm PMMA particle as shown in Fig. S5. The MIP signal from the PMMA particle originates from the scattering intensity modulation as the size and refractive index change due to heating. Such scattering field change per kelvin can be evaluated by utilizing the Mie-scattering theory for given material and size as introduced in the reference[2]. For the backscattering of D=500 nm PMMA particle with a collection NA of 1.2, the average change of scattering intensity is 0.39%/K. With the raw PDI signal acquired (Fig. S5a), we can calculate modulation depth by dividing the photothermal amplitude with concurrently acquire scattering intensity amplitude (particle intensity subtract background intensity). The corresponding modulation depth and estimated temperature rise is shown in Fig.S5b. For this D=500nm PMMA particle under mid-IR excitation at 1729 cm^{-1}, the measured modulation depth was ~3% and the highest temperature rise was 7.6 K.

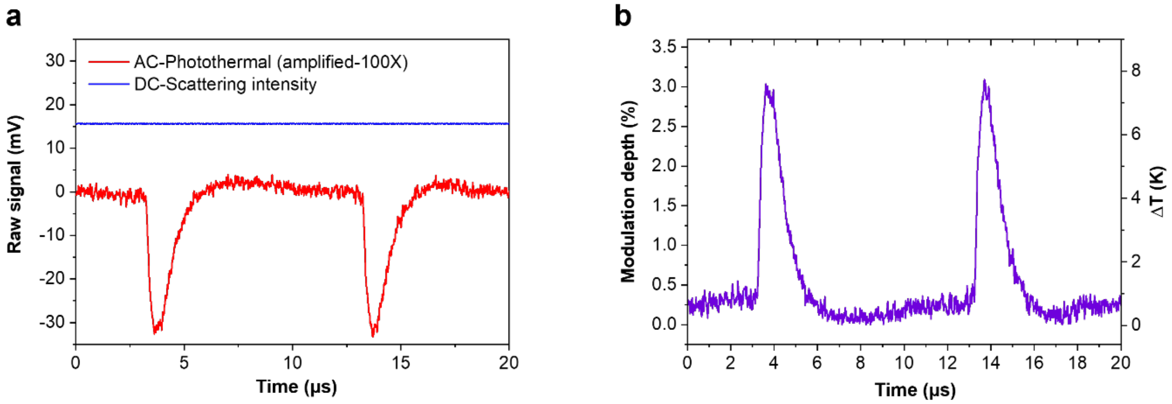

Supplementary Figure 5. Transient temperature rising of D=500 nm PMMA particle under mid-IR excitation at 1729 cm^{-1}. (a) Raw PDI signal from D=500 nm PMMA particle. Raw photocurrent signal is separated into DC and AC components. AC is amplified 100 times. DC is directly digitized synchronically. (b) Modulation depth and calculated temperature rise from signal shown in (a). The modulation depth is calculated by dividing the AC signal amplitude with pure DC scattering intensity amplitude and amplification. Transient temperature is derived by diving modulation depth with 0.39%/K, the average scattering intensity change percent per kelvin evaluated from Mie-scattering according to the method in reference[2]. The peak modulation depth is 3% according to the highest temperature rise of 7.6 K.

Supplementary Note 4 | Complex photothermal signal from lipid droplets

The heat transfer model used for photothermal dynamics analysis in the main text assumes that the environmental temperature around the absorber is constant. This assumption holds for most cases that a small absorber is immersed in a bulky medium (e.g. isolated particle in air, water), where the temperature rise of the ambient environment is negligible. However, the temperature of the microenvironment change ΔT_{env} outside the probe beam focal spot is more likely to be influenced by the presence of medium absorption and complex heat dissipation from surrounding absorbers. In such cases, the heat dissipation process may consist of multiple exponential decays with distinct time constants. This multiple decay model can be derived by changing the expression of heat dissipation power Q_{diss} to:

$$Q_{\mathrm{diss}} = \theta_1[T(t) - T_{\mathrm{env}}(t)] \tag{6}$$

in which, the temperature of microenvironment $T_{\mathrm{env}}(t)$ is modified to a time-dependent variable. θ_1 is the heat dissipation coefficient of the microenvironment to its surrounding with the unit of W/K. More specifically, $T_{\mathrm{env}}(t)$ can also be described with Newton's law:

$$C_{\mathrm{env}} \frac{\mathrm{d}T_{\mathrm{env}}}{\mathrm{d}t} = \theta_2[T_{\mathrm{env}}(t) - T_0(t)] \tag{7}$$

Where C_{env} is the heat capacity of the microenvironment, θ_2 is the heat dissipation coefficient of the microenvironment to its surrounding with the unit of W/K. $T_0(t)$ is the ambient temperature outside the microenvironment. The solution for the above differential equation has the form of summation of multiple exponential functions with different time constants, thus the temperature evolution of the sample inside probe focal spot has the form:

$$T(t) = \Sigma_i^n A_i e^{-t/\tau_i} \tag{8}$$

The above form indicates a complex decay signal composed of multiple lifetimes. This phenomenon is experimentally discovered in the lipids droplets inside a cell as shown in Fig.S6. Those photothermal dynamics signals show a decay with at least two distinct lifetimes. The shorter one is below 500 ns, like the single lipid droplets decay shown in Fig.5d. On the contrary, the second lifetime is much longer in the microsecond level, representing a relatively slow environmental temperature evolution from the surrounding microenvironment.

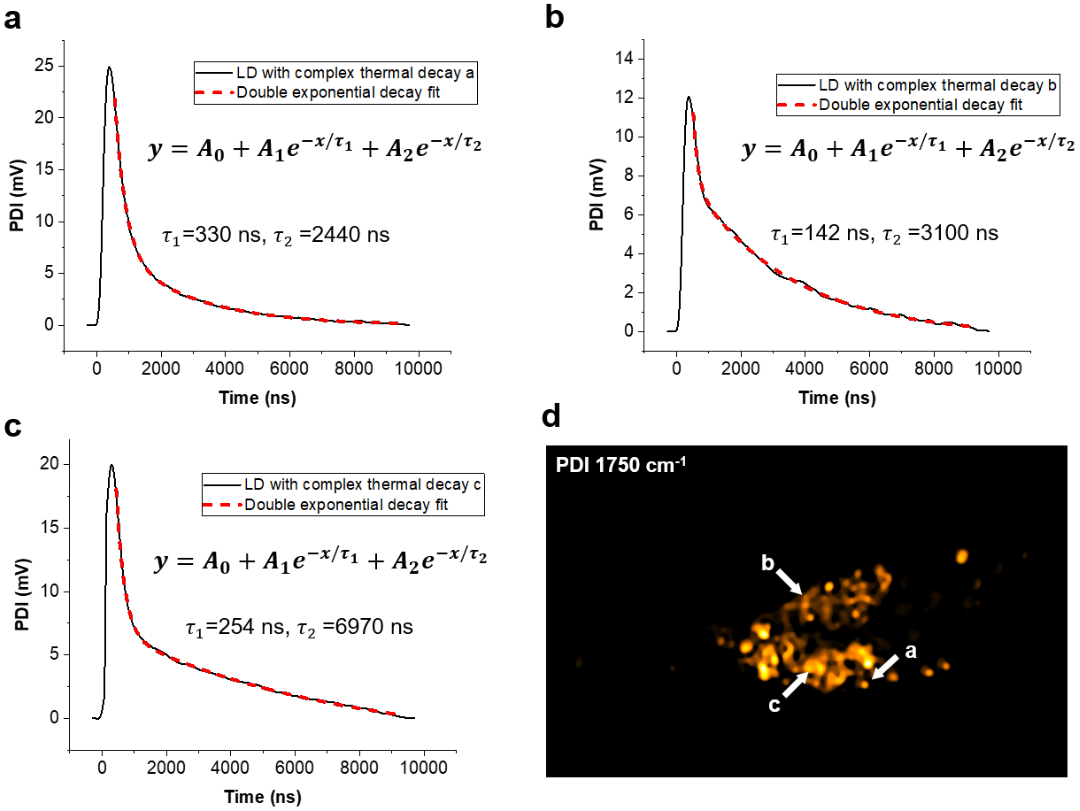

Supplementary Figure 6. Complex decay signal with multiple time constants τ and their positions in cell.

	Lock-in	Boxcar	PDI
Output contrast	Amplitude at selected harmonics; up to 8 channels	Amplitude difference between on-off state	Complete temporal dynamics of modulation
Dynamics information	Phase delay to the reference;<br>up to 8 channels	Temporal dynamics can be acquired by scanning the gating delay	Comprehensive decay information acquired at once
Preferred input signal for high SNR	50% duty cycle	Low duty cycle (<50%)	No requirements, versatile processing methods to match different signals
User end signal processing	Not required	Not required	Required
Input bandwidth	600MHz (1.8GHz digitizer inside)	600MHz (1.8GHz digitizer inside)	Half of digitizer sampling rate (Up to 2GHz)
Maximum channel number	2-channel	2-channel	128-channel or more for high freedom in customization

Supplementary Table 1: Comparison of state of art lock-in, boxcar and PDI for temporal dynamics detection

Supplementary references

1 Bergman, T. L., Incropera, F. P., DeWitt, D. P. & Lavine, A. S. *Fundamentals of heat and mass transfer*. (John Wiley & Sons, 2011).

2 Li, Z., Aleshire, K., Kuno, M. & Hartland, G. V. Super-resolution far-field infrared imaging by photothermal heterodyne imaging. *The Journal of Physical Chemistry B* **121**, 8838-8846 (2017).
