# Supplementary material for: Nanosecond-resolution photothermal dynamic imaging via MHZ digitization and match filtering
Source: Nat Commun. 2021 Dec 7;12:7097. doi: 10.1038/s41467-021-27362-w (PMC8651735; doi:10.1038/s41467-021-27362-w)
Supplement: Supplementary file 3 — Description of Additional Supplementary Files [file 41467_2021_27362_MOESM3_ESM.docx]

**Description of Additional Supplementary Files**

**Supplementary Movie 1.** Time resolved PDI of 300-nm diameter PMMA particles

**Supplementary Movie 2.** Time resolved PDI of mixture of 300-nm and 500-nm diameter PMMA particles
